# Serum *survivin* predicts responses to treatment in active rheumatoid arthritis: a post hoc analysis from the SWEFOT trial

**DOI:** 10.1186/s12916-015-0485-2

**Published:** 2015-09-30

**Authors:** Adrian Levitsky, Malin C. Erlandsson, Ronald F. van Vollenhoven, Maria I. Bokarewa

**Affiliations:** Unit for Clinical Therapy Research, Inflammatory Diseases (ClinTRID), Karolinska Institutet, D1:00, Karolinska University Hospital, 17176 Stockholm, Sweden; Department of Rheumatology and Inflammation Research, Sahlgrenska University Hospital, University of Gothenburg, Gothenburg, Sweden

**Keywords:** Rheumatoid arthritis, Biomarkers, *Survivin*, Methotrexate, Disease-modifying antirheumatic drugs, Anti-TNF, Disease activity, Functional disability

## Abstract

**Background:**

The identification of biomarkers that predict optimal and individual choices of treatment for patients with rheumatoid arthritis gains increasing attention. The purpose of this study was to investigate if the proto-oncogene *survivin* might aid in treatment decisions in early rheumatoid arthritis.

**Methods:**

Serum *survivin* levels were measured in 302 patients who completed the Swedish pharmacotherapy (SWEFOT) trial at baseline, 3, 12, and 24 months. *Survivin* levels > 0.45 ng/mL were considered positive. Based on the *survivin* status, core set outcomes measuring disease activity, functional disability, as well as global health and pain were evaluated after methotrexate (MTX) monotherapy at 3 months, and at 12 and 24 months of follow-up. Treatment of non-responders was randomly intensified with either a combination of disease-modifying antirheumatic drugs (triple therapy: MTX, sulfasalazine, and hydroxychloroquine) or by adding antibodies against tumor necrosis factor (anti-TNF).

**Results:**

Antirheumatic treatment resulted in an overall decrease of serum *survivin* levels. *Survivin*-positive patients at baseline who initially responded to MTX had a higher risk of disease re-activation (OR 3.21 (95 % CI 1.12–9.24), *P =* 0.032) and failed to improve in their functional disability (*P =* 0.018) if having continued on MTX monotherapy compared to *survivin*-negative patients. Ever-smokers who were *survivin*-positive were less likely to respond to MTX than those who were *survivin*-negative (OR 1.91 (1.01–3.62), *P =* 0.045). In *survivin*-positive patients, triple therapy led to better improvements in disease activity than did MTX + anti-TNF. At 24 months, *survivin*-positive patients randomized to anti-TNF had a higher risk of active disease than those randomized to triple therapy (OR 3.15 (1.09–9.10), *P =* 0.037).

**Discussion:**

We demonstrate for the first time that *survivin *is a valuable serologic marker that can distinguish drug-specific clinical responses in early rheumatoid arthritis through the pragmatic clinical setting of the care-based SWEFOT trial. Although treatment response cannot solely be attributable to *survivin *status, per protocol sensitivity analyses confirmed the superior effect of triple therapy on *survivin*-positive patients.

**Conclusions:**

*Survivin*-positive patients have poor outcomes if treated with MTX monotherapy. A decrease of *survivin* levels during treatment is associated with better clinical responses. For *survivin*-positive patients who fail MTX, triple therapy is associated with better outcomes than anti-TNF therapy.

**Trial registration:**

WHO database at the Karolinska University Hospital: CT20080004; ClinicalTrials.gov: NCT00764725, registered 1 October 2008.

## Background

Despite advances in antirheumatic treatment, rheumatoid arthritis (RA) continues to be associated with a significant disease burden due to reduced quality of life [1], functional disability [2], and an enhanced prevalence of comorbidities [3, 4]. Marked individual heterogeneity with respect to specific genetic and environmental load, autoantibody production, and cellular, cytokine and gene expression profiles of the inflamed synovia strongly suggests that a more personalized choice of antirheumatic treatment would yield considerably better results. A requirement of biomarkers and algorithms capable of predicting treatment response and reducing unnecessary expenses on the costs of inefficient medication has recently been given high priority [5].

Methotrexate (MTX) is recommended as the top first-line antirheumatic drug owing to its relatively high efficacy and low rate of adverse events [6]. The combination of MTX with glucocorticoids [7], other conventional antirheumatic drugs [8], or with inhibitor of tumor necrosis factor (anti-TNF) [9, 10] could be more effective than MTX monotherapy. For patients who fail initial MTX therapy several reasonable options are available, including the addition of sulfasalazine (SSZ) and hydroxychloroquine (HCQ) – the so-called ‘triple therapy’ (TT) – or the addition of anti-TNF. The Swedish pharmacotherapy (SWEFOT) trial was a standard care-based study [11], where all patients initially received open-label MTX monotherapy followed by randomized treatment with either TT or with MTX in combination with anti-TNF (in this case, infliximab) in the patients who failed to achieve low disease activity on MTX. The trial showed that anti-TNF was clinically superior to TT after 12 months, while the difference between the treatment arms leveled-off after 24 months [12]. Recent randomized controlled trials, which compared the combination of conventional disease-modifying drugs with biologic strategies, demonstrated that both treatment options can be successful for some patients [13, 14]. The results of randomized and observational studies [15, 16] indicated a substantial difference in social care costs for these treatment alternatives. Thus, exponential increases in expenses urge reliable indicators that would predict the optimal treatment choice for every patient.

The proto-oncogene *survivin* is a biomarker of cancer and may be found in most tumor tissues, such as lymphoma, colorectal carcinoma, breast cancer, small cell lung adenocarcinoma, and others [17–20], where it predicts prognosis and the potential for metastasis. Cellular functions of *survivin* comprise inhibition of apoptosis in the cytoplasmic and mitochondrial compartments by preventing activation of caspases, and regulation of the cell cycle progression in the nucleus by aiding formation of a chromosomal passenger complex [21, 22]. In healthy tissues, *survivin* expression is indispensable for cell renewal and differentiation, being consistently expressed in thymocytes, bone marrow hematopoietic progenitors and stem cells, cells of the colon epithelium, and vascular endothelial cells [23–25].

In RA, serum *survivin* has recently emerged as a marker of the disease. It is over-expressed in the pre-clinical phase of RA, and, together with antibodies to citrullinated peptides, is predictive for development of RA several years ahead of clinical symptoms [26]. Importantly, in the pre-symptomatic stage of RA, *survivin* was associated with the pattern of regulatory cytokines (interleukin (IL)-12, IL-1, IL-9, granulocyte-macrophage colony-stimulating factor, and IL-2) controlling the formation of pathogenic T helper (Th) 1 and Th17 lymphocytes. Also, *survivin* has been recently connected to carriage of the human leukocyte antigen (HLA) DRB1 genotype and smoking [27, 28], important keystones in the pathogenesis of RA. *Survivin* is critical for the process of antigen presentation – the breaking point of immune responses in RA, being required for the expression of major histocompatibility complex class II molecule receptors on dendritic cells [29] and for the formation of functional T cell receptors [30, 31]. Expression of *survivin* in B cells might be attributable to adverse cell recognition in RA, since changes in *survivin* expression after therapeutic B cell depletion was associated with a reduction of B cell numbers, serum levels of rheumatoid factor (RF) and the activity of arthritis [32]. In observational study cohorts, *survivin* assists with the early recognition of RA patients with poor prognosis, being associated with progressive joint damage and a low rate of treatment response [33–35]. The role of *survivin* as a clinical predictor of drug-specific treatment response has not been investigated in RA. Therefore, the design of the SWEFOT trial provided an opportunity for the simultaneous evaluation of clinical outcomes of different antirheumatic treatment strategies with respect to the *survivin* status of the patients. In this post hoc analysis we asked if high levels of *survivin* in serum identified RA patients with poor response to antirheumatic treatment and worse clinical outcomes over time.

## Methods

### Study design

**Fig. 1:**
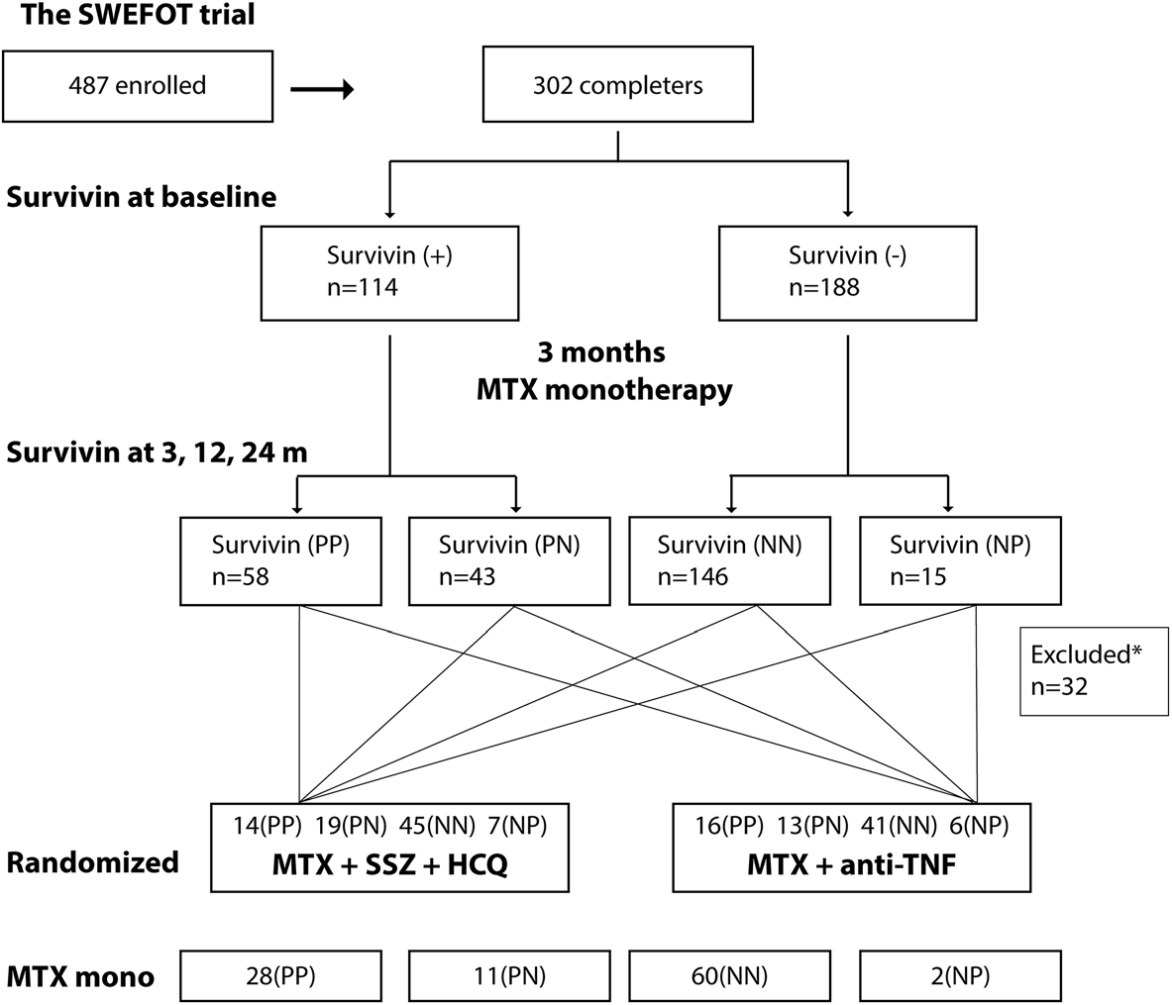
Analysis profile. Serum levels of *survivin* were measured from samples of 302 patients who completed the 24-month follow-up of the SWEFOT trial. Measurements at baseline resulted in the *survivin*-positive (> 0.45 ng/mL) and *survivin*-negative patient groups. Measurements at 3, 12, and 24 months identified patients who decreased or increased their serum *survivin* levels at any time point over 24 months. Four groups of patients were formed and compared: patients positive for *survivin* (PP), or negative for *survivin* (NN) on all testing occasions; and patients *survivin*-positive at baseline who converted negative (PN), or *survivin*-negative patients who converted positive (NP). After 3 months of methotrexate (MTX) monotherapy, patients still with active disease were randomized to triple therapy (MTX + sulfasalazine (SSZ) + hydroxychloroquine (HCQ)) or to anti-TNF (MTX + infliximab). *Patients with missing samples at 12 and 24 months (n = 15), who were not randomized (n = 2), or who changed their *survivin* status on several occasions (n = 15) were excluded from analysis

The SWEFOT trial is an open-label randomized study comparing treatment strategies in patients with early RA [12]. Patients from 15 rheumatology units in Sweden with symptom duration < 1 year and previously not treated with disease-modifying antirheumatic drugs were invited to participate in this trial. At inclusion, 487 patients with the 28-joint count disease activity score (DAS28) > 3.2 were enrolled in the trial between December 2002 and 2006 (Fig. 1). All patients were initially treated with methotrexate (MTX, 20 mg/week). Clinical assessment at 3 months distinguished patients with DAS28 ≤ 3.2 (MTX responders) and DAS28 > 3.2 (MTX non-responders). MTX responders continued treatment with MTX monotherapy, while MTX non-responders were randomized to TT (MTX + SSZ + HCQ) or to anti-TNF therapy (MTX + infliximab). Clinical assessment of the patients was performed at baseline and thereafter at 3, 12, and 24 months using the DAS28 and other ‘core set’ outcomes, including functional disability measured by the Health Assessment Questionnaire (HAQ), pain perception graded by visual analog scale (pain-VAS), and patient’s global assessment of disease activity (PtGA-VAS). A total of 302 patients completed the 24-month trial period by intention-to-treat and were the subjects for this analysis.

The study protocol was approved by the regional ethical committees of Sweden. The SWEFOT trial has registration identification CT20080004 at the World Health Organization (WHO) database at the Karolinska University Hospital, Stockholm, and registration identification NCT00764725 at ClinicalTrials.gov. At enrollment, all patients gave their written informed consent to participate in the study.

### Measurements of serum *survivin* levels

Blood samples were obtained at baseline before MTX treatment, and at 3 months for all patients enrolled in the SWEFOT trial. At 12 and 24 months, samples for the MTX non-responders were available. Samples were centrifuged and stored at −80 °C.

Serum *survivin* levels were measured using a matched-antibody pair (rabbit anti-human *survivin*) by a sandwich enzyme-linked immunosorbent assay (DYC647, R&D Systems, Minneapolis, MN, USA) [33, 36]. The detection limit of the assay was 0.1 ng/mL. The cut-off level of 0.45 ng/mL was set as previously reported [29], and was used to distinguish between *survivin*-positive (+) and *survivin*-negative (−) patients.

### Statistical analysis

The statistical analyses were based on intention-to-treat, last observation carried forward. Changes in *survivin* status after 3 months of MTX treatment resulted in the formation of four *survivin* groups: *survivin*-positive (+/+); *survivin*-negative (−/−); *survivin* converting to negative (+/−); and *survivin* converting to positive (−/+). Patients who changed *survivin* more than once (n = 15) were excluded from the analysis.

Utilizing IBM SPSS v.22.0, and OpenEpi.com [37], nonparametric statistical comparisons were performed. Data is presented as the median and interquartile range (IQR) for continuous variables, and frequency (%) or odds ratio (OR) (95 % confidence interval, CI) for proportions. Mann–Whitney U tests and Wilcoxon signed-rank tests were used to compare continuous variables, whereas Pearson’s *χ*^2^ or Fisher’s exact tests were utilized for proportions. For the comparison of more than two groups, initial analysis was done by Kruskal–Wallis tests, followed by pairwise post hoc analyses by Dunn-Bonferroni correction. All tests were two-tailed and performed at the 0.05 level of significance.

## Results

### Changes of serum *survivin* levels during the SWEFOT trial

**Fig. 2:**
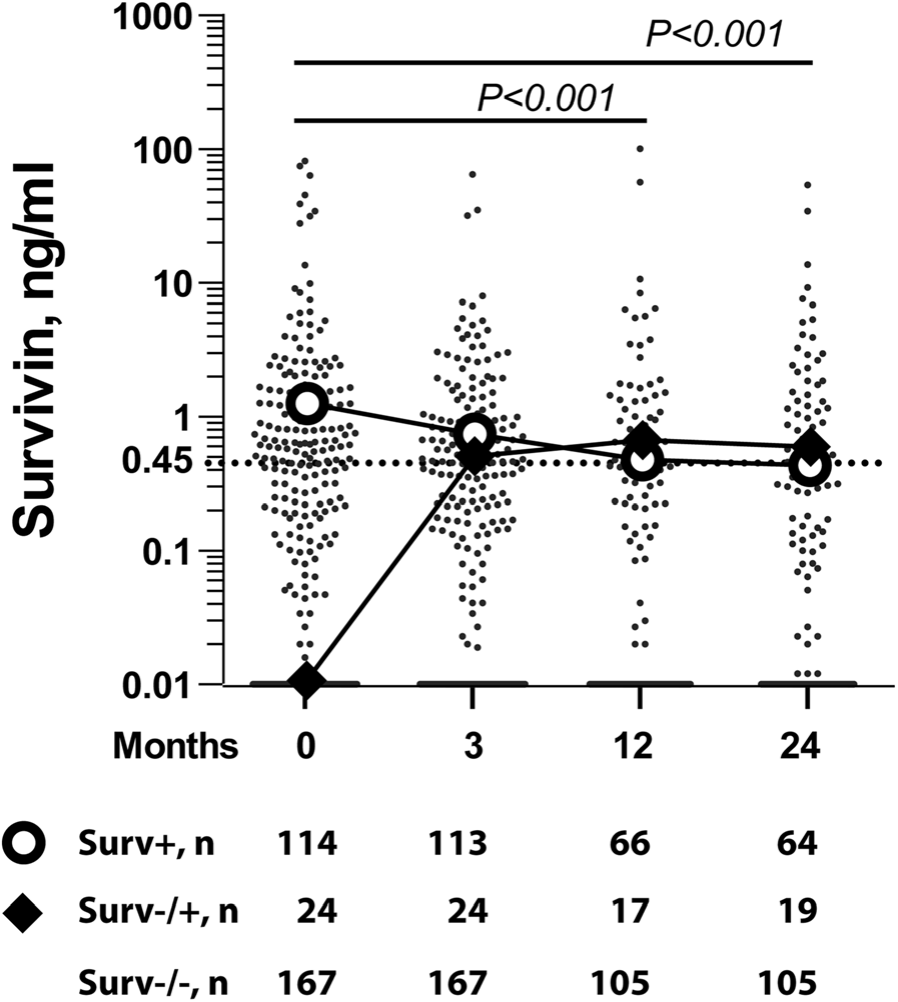
Changes of serum *survivin* levels during the SWEFOT trial. Serum levels of *survivin* were measured in 302 patients enrolled in the trial at baseline, where 114 patients were *survivin*-positive (*survivin* > 0.45 ng/mL, dashed line), and the remaining 188 patients were *survivin*-negative. In total, a decrease of *survivin* levels was observed at 3, 12, and 24 months of antirheumatic treatment. The number of samples available for the analysis is indicated at each time point. Bolded lines indicate median levels of the *survivin*-positive at baseline group (open circles) and the *survivin*-negative converting to positive group at any time point over 24 months (filled rhombi). A total of 52 (46 %) of the *survivin*-positive patients converted to negative over 24 months. Comparison between the time points was done by the Wilcoxon signed-rank test, and the *P* values for the total patient cohort are indicated

At baseline, 114 of 302 patients (38 %) were *survivin*-positive (+). The *survivin* (+) patients were significantly more often RF (+) and tended to have higher functional disability by HAQ compared to *survivin* (−) patients (Table 1). There were no differences in baseline disease activity by DAS28 or any other core set clinical outcomes. Serum *survivin* levels decreased significantly from baseline over 24 months (Fig. 2). The majority of patients (167/294, 56.8 %) remained *survivin*-negative (−/−) by 3 months, and 75 patients remained *survivin*-positive (+/+). Thirty-eight patients converted to negative (+/−) by 3 months and an additional 14 patients converted to negative (+/−) by 24 months. Of the initially *survivin* (−) patients, 24/188 (12.8 %) became *survivin*-positive (−/+) at any time point over 24 months (Fig. 2). The clinical significance of the change in *survivin* levels with respect to the core set outcomes was assessed for the *survivin* (+/+), (+/−), (−/−), and (−/+) groups (Fig. 1).

**Table 1:** Baseline clinical characteristics of patients with early RA enrolled into SWEFOT, divided by *survivin* status

Variables	*Survivin*-positive	*Survivin*-negative	*P* value
	n = 114	n = 188	
Age (years)	56.0 (43.0, 62.25)	57.0 (44.0, 67.0)	0.332
Sex (F)	77 (68 %)	143 (76 %)	0.107
Duration	5.0 (4.0, 8.0)	5.0 (4.0, 8.75)	0.905
RF (+)	91/113 (81 %)	108/187 (58 %)	<0.001
Anti-CCP (+)	71/107 (66 %)	103/183 (56 %)	0.091
Pain-VAS	60.0 (45.75, 72.0)	54.0 (39.0, 71.0)	0.269
PtGA-VAS	60.0 (39.0, 77.0)	58.0 (35.25, 74.0)	0.452
HAQ	1.25 (0.85, 1.75)	1.0 (0.75, 1.5)	0.079
TJC	8.0 (5.0, 13.0)	9.5 (6.0, 14.0)	0.134
SJC	11.0 (6.0, 14.0)	10.0 (7.0, 14.0)	0.692
ESR	35.0 (21.5, 63.0)^a^	34.0 (19.25, 50.0)	0.260
CRP	17.0 (9.0, 54.5)^a^	18.0 (9.0, 37.0)	0.628
DAS28	5.78 (5.06, 6.35)^a^	5.72 (5.02, 6.43)	0.751

^a^Number of patients, n = 113. Serum levels of *survivin* >0.45 ng/mL indicate *survivin*-positive patients. Statistics in the groups are presented as medians and interquartile range (IQR). Comparisons between the groups were done by Mann–Whitney U tests for continuous variables, and by Pearson’s *χ* ^2^ tests for frequencies. Anti-CCP, antibodies to cyclic citrullinated peptides; CRP, C-reactive protein; DAS28, 28-joint count disease activity score; ESR, erythrocyte sedimentation rate; F, females; HAQ, Health Assessment Questionnaire; pain-VAS, visual analog scale for pain; PtGA-VAS, patient’s global assessment of disease activity; RA, rheumatoid arthritis; RF, rheumatoid factor; SJC, swollen joint count; TJC, tender joint count

### Clinical outcomes with MTX monotherapy

After 3 months of MTX treatment, there were 101 MTX responders and 193 MTX non-responders with available *survivin* status. As reported, the MTX responders had significantly better treatment outcomes compared to non-responders with respect to DAS28 and other clinical outcomes (*P* < 0.001) [11, 12, 38]. Thirty-nine of 101 (38 %) MTX responders were *survivin* (+), and so were 74 of 193 (38 %) MTX non-responders. Since high levels of *survivin* were associated with smoking [27, 39], we analyzed if the response to treatment was dependent on the smoking habits of the patients. The prevalence of ever-smokers was similar among *survivin* (+) (68 %) and (−) (61 %) patients at baseline. Among the ever-smokers, *survivin* (+) patients had a higher probability to be MTX non-responders compared to *survivin* (−) patients (51/71 versus 64/112, OR 1.91 (95 % CI 1.01–3.62), *P =* 0.045). *Survivin* conversion to negative (+/−) occurred without a difference between ever- and never-smokers (23/165 versus 13/96).

**Fig. 3:**
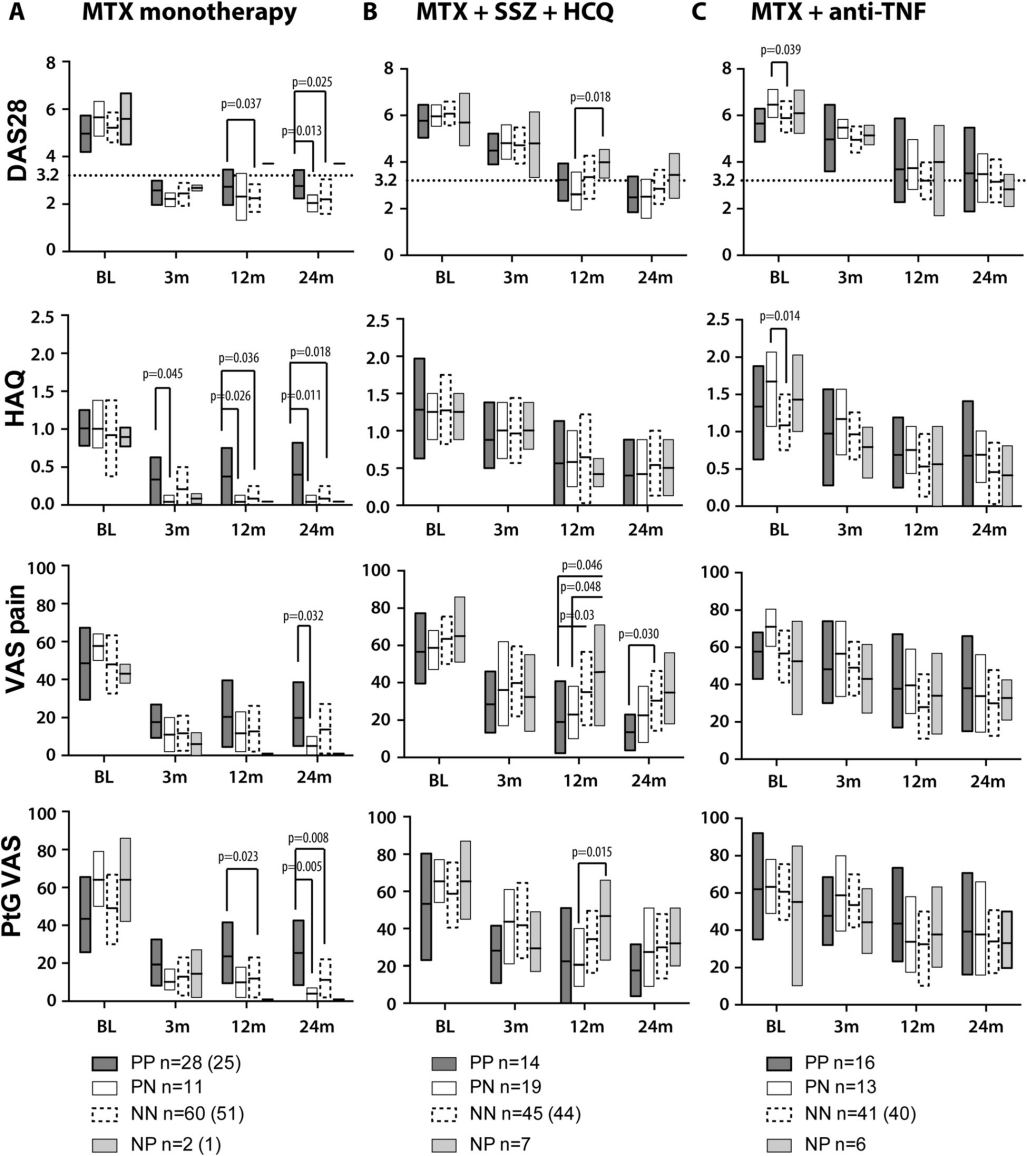
Core set clinical parameters in the *survivin* subgroups of the SWEFOT trial. At baseline, all patients were treated with methotrexate (MTX). **a** At 3 months, the patients with a disease activity score (DAS28) < 3.2 proceeded on MTX monotherapy. **b** The patients with a DAS28 > 3.2 were randomized to triple therapy with MTX + sulfasalazine (SSZ) + hydroxychloroquine (HCQ), **c** or to anti-TNF therapy (MTX + infliximab). The intention-to-treat analysis was conducted for clinical parameters, including the DAS28, functional disability (Health Assessment Questionnaire, HAQ), pain perception with the visual analog scale (pain-VAS), and the patient’s global assessment of disease activity (PtGA-VAS). Boxes represent the 25th to 75th percentile of the group, and horizontal lines within the boxes indicate median values. Four groups of patients were compared: patients positive for *survivin* (PP), and negative for *survivin* (NN) at baseline and over 24 months; patients positive for *survivin* at baseline who converted negative (PN), and negative for *survivin* who converted positive (NP) over 24 months. Comparisons of the absolute values were done by Mann–Whitney U tests

At 3 months, the *survivin* (+/+) MTX responders had a higher HAQ compared to those who converted negative (+/−), indicating a delay in improvement of their functional disability (Fig. 3a). This higher HAQ was not directly related to the disease activity, since the *survivin* (+/+) patients had no significant differences in DAS28, pain-VAS, and PtGA-VAS at 3 months when compared to the other *survivin* groups.

Among initial MTX responders who continued on MTX monotherapy, *survivin* (+) patients had a higher risk of disease reactivation (DAS28 > 3.2) at 12 months compared to *survivin* (−) patients (12/36 versus 7/52, OR 3.21 (1.12–9.24), *P =* 0.032). Also, *survivin* (+/+) patients on MTX monotherapy had significantly higher DAS28, HAQ, and PtGA-VAS compared to the *survivin* (−/−) patients (Fig. 3a). This difference was maintained at 24 months compared to the *survivin* (+/−) and (−/−) subgroups (Fig. 3a).

### Clinical outcomes with combined antirheumatic treatments

MTX non-responders were randomized to TT (n = 85) or to anti-TNF (n = 76) treatment. At 12 months, the TT-treated *survivin* (+/−) subgroup (n = 19) had better outcomes compared to the *survivin* (−/+) subgroup (n = 7) in DAS28, pain-VAS and in PtGA-VAS (Fig. 3b), and had significantly lower DAS28 and pain-VAS compared to anti-TNF-treated *survivin* (+/−) patients (n = 13) (median (IQR) 2.34 (1.94, 3.56) versus 3.43 (2.82, 4.96), *P =* 0.045; and 21.0 (10.0, 38.0) versus 35.0 (24.5, 59.0), *P =* 0.033, respectively). Among anti-TNF-treated patients, the *survivin* subgroups had no differences in core set outcomes (Fig. 3c).

**Fig. 4:**
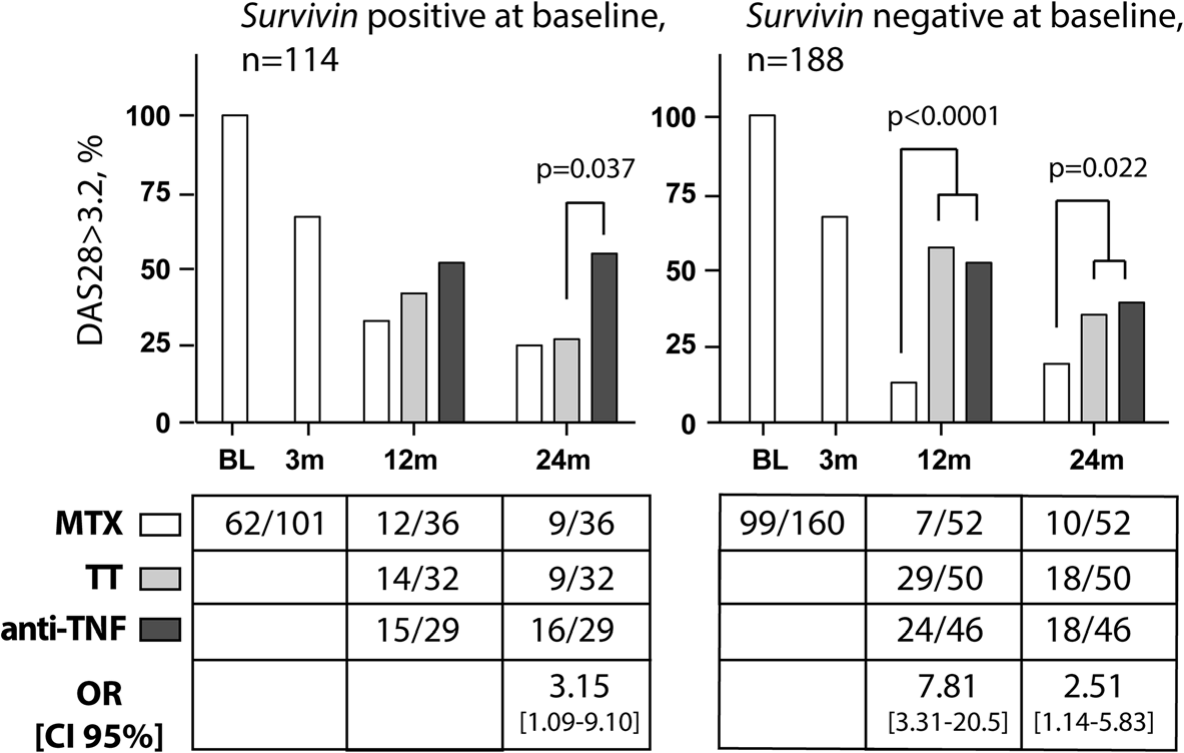
Prevalence of active disease among *survivin*-positive or *survivin*-negative patients in the SWEFOT trial. The prevalence of active disease (disease activity score, DAS28 > 3.2) among patients who were *survivin*-positive or *survivin*-negative at baseline is presented for the groups treated with methotrexate (MTX) monotherapy and the groups randomized to triple therapy (TT) or to anti-TNF therapy. Comparisons were done by Pearson’s *χ* ^2^ or Fisher’s exact tests, and odds-based estimates (odds ratio, OR) and 95 % confidence intervals (CI) are indicated. Two patients with no available DAS28 at 3 months were excluded from the analysis

At 24 months, better clinical outcomes were found among the *survivin* (+) patients at baseline ((+/+) and (+/−), n = 14 + 19) randomized to TT compared to the *survivin* (+) patients (n = 16 + 13) randomized to anti-TNF. The TT-treated *survivin* (+) patients attained a significantly lower DAS28 (2.37 (1.79, 3.27) versus 3.50 (2.05, 4.63), *P =* 0.020), and the estimated risk of active disease activity (DAS28 > 3.2) was higher among the anti-TNF-treated *survivin* (+) patients (55 % versus 28 %; OR 3.15 (95 % CI 1.09–9.10), *P =* 0.037) (Fig. 4). Consequently, the prevalence of DAS28 *<* 3.2 among the TT-treated *survivin* (+) patients was similar to MTX responders (72 % and 75 %, respectively). Analogously, TT-treated *survivin* (+/+) patients (n = 14) had a lower pain-VAS compared to anti-TNF-treated *survivin* (+/+) patients (n = 16) (14.0 (3.75, 23.0) versus 33.0 (15.0, 66.0), *P =* 0.038), and the *survivin* (+/−) patients treated with anti-TNF had a high frequency of active disease compared to the TT-treated subgroup (62 % versus 26 %, *P =* 0.046). *Survivin* (−/−) patients showed similar responses to TT and anti-TNF treatment and reached comparable DAS28, HAQ, pain-VAS, and PtGA-VAS outcomes (Fig. 3b,c). The proportion of patients with DAS > 3.2 among MTX responders and *survivin* (−) at baseline ((−/−) and (−/+), n = 51 + 1) was always lower when compared to *survivin* (−) MTX non-responders (n = 83 + 13) (Fig. 4). The *survivin* (−/+) patients treated with TT and anti-TNF had similar outcomes.

## Discussion

We studied the importance of serum *survivin* levels during antirheumatic treatment by post hoc analysis of the SWEFOT trial cohort. Our results confirm that patients *survivin*-positive at baseline can have poor long-term outcomes. Patients who remain positive by 3 months, despite initially responding by DAS28, are recognized by a risk for later disease reactivation and possess early functional disability that deteriorates even further over 24 months. These findings support the previously reported association between radiographic damage and a considerable gain in HAQ over time despite clinical remission [40]. It also may at least in part explain the observations from the BARFOT cohort indicating significantly higher 60-month disease activity and radiographic progression in *survivin*-positive early RA patients [27]. Notably, the *survivin*-positive patients within the BARFOT and SWEFOT cohorts of early RA patients had comparable levels of DAS and HAQ at 24-month follow-up despite obvious differences in therapeutic choices and significantly higher remission rates reached by groups with different *survivin* status within the SWEFOT trial.

Female gender, age, current smoking, and functional disability as predictors of MTX response were previously established [41]. We extend the list of predictors with *survivin* measurements and show that *survivin* binds together as an environmental risk represented by smoking, with clinical parameters of disease activity and functional disability. Concerning the analysis of *survivin* groups, neither the presence of RF or antibodies against citrullinated peptides, nor the combined multi-biomarker disease activity score [42] supported discrimination in the disease outcome achieved by *survivin* measurements (results not shown). Taken in concert, these observations confirm the independent predictive value of *survivin* measurements in early RA and suggest that *survivin*-positive patients require initial combination treatment to avoid potentially irreversible outcomes.

The other important finding of this study is related to the choice of treatment after failure to respond adequately to MTX. The primary response to MTX and early remission remained a major predictor of long-term clinical outcomes for patients in the SWEFOT trial, and the combined intensive treatment for MTX non-responders in this trial was not sufficient for achieving the same outcomes. The importance of MTX response has been initially reported in randomized goal-steered treatment studies [43, 44] and repeated by observational studies [45, 46] and a meta-analysis [47]. Our data show that for *survivin*-positive patients, TT is an effective treatment option with a rate of remission in this group comparable to the MTX responder group. Surprisingly, anti-TNF treatment appeared to be less successful for *survivin*-positive MTX non-responders.

Several approaches to understanding the nature of poor anti-TNF response have been proposed, where genetic variations and TNF-independent mechanisms of arthritis have been extensively explored. Despite extensive candidate gene-driven and whole-genome based research, presenting several loci involved in anti-TNF treatment response, no sustainable solutions have currently been found [48–50]. In addition, attempts to identify autoantibody and cytokine profiles suggest a potential predictive signature [42, 51, 52], although, in light of these promising results, the practical value remains yet to be elucidated.

The SWEFOT trial demonstrates that *survivin* is a changeable serologic marker with a notable connection to disease outcome. A decrease of serum *survivin* levels is shown to be an overall consequence of antirheumatic treatment, and serological conversion to *survivin*-negative occurred in about a half of the *survivin*-positive patients. Most of the *survivin* conversion was identified after 3 months of MTX monotherapy. It was coupled with excellent clinical outcomes and a low reactivation rate at follow-up. In contrast, patients who gained *survivin*-positivity were comprised almost solely of MTX non-responders with poor outcomes, irrespective of treatment modality. For the first time, *survivin* status and conversion have been associated with clinical manifestations of early RA in a pragmatic clinical setting of the broad care-based SWEFOT trial. In the original report [11], the anti-TNF group was shown to provide significantly better clinical responses and non-significantly different radiographic outcomes in comparison to TT at 12 months, which was reversed at 24 months [12]. Now, we identify a subgroup of *survivin*-positive patients not responding effectively to anti-TNF therapy. Thus, the monitoring of *survivin* levels assists in prognosis and treatment decisions for patients with early RA.

The nature of extracellular *survivin* release remains an enigma. Since active extracellular transport of *survivin* is described only as exosomal content [53], profound cellular disruption could be a cause of intermittent serum levels of *survivin*. The poor response to anti-TNF treatment observed in this study and lack of a direct correlation between serum *survivin* and inflammatory markers, including C-reactive protein and IL-6 [29, 33, 54], suggests a TNF-independent mechanism of *survivin* release. Extracellular *survivin* has been shown to be biologically active inducing surface expression of adhesion molecules on leukocytes of RA patients [55] with a potential to regulate T cell functions and motility through a broad net of intracellular effectors [56, 57]. At the preclinical stage of arthritis, serum *survivin* has been associated with the release of cytokines controlling the formation of Th cell subsets Th1 and Th17 [26, 58]. Inhibition of *survivin* in experimental arthritis proved its intimate relation to the formation of effector T cells and to the system of matrix proteases in the inflamed joints [54, 59]. The processes triggering and abrogating *survivin* release in RA could therefore pave a way to efficient therapeutic control of the disease.

Several points in the specificity of this analysis could have influenced the results. The SWEFOT trial does not account for patients changing treatment by adjusting drug doses or switching to cyclosporine A for the TT group or etanercept for the anti-TNF group due to toxicity. Drug cytotoxicity could be responsible for disease-independent *survivin* release, although the number of such patients was small. Also, a group of patients with frequently changeable *survivin* status were excluded from statistical analysis. The analysis does not account for the autoantibody status within the *survivin* groups, since no association between the presence of autoantibodies and a decrease of *survivin* levels was noted after 3 months of MTX monotherapy. Combined *survivin* and autoantibody analysis could have strengthened the obtained results due to the tight coexistence of these biomarkers in severe RA [27, 28]; however, it could have given the opposite result due to the association of autoantibodies with a strong response to anti-TNF treatment [52]. The limited final numbers of patients allocated and followed-up within the TT- and anti-TNF-treated groups do not permit a complete exclusion of a serendipitous outcome. Nonetheless, the SWEFOT study was adequately powered to address whether *survivin* could be a predictor of response, with the utilization of robust non-parametric statistical methods – and sensitivity analyses of the patients who completed the study per protocol confirmed the superior effect of TT on *survivin*-positive patients. Finally, it is important to keep in mind that poor outcomes among the groups of MTX non-responders are not restricted to *survivin* status. A substantial number of *survivin*-negative patients failed to respond to TT and anti-TNF treatment for reasons yet unknown.

## Conclusions

Our results indicate that the measurement of serum *survivin* is useful for planning treatment strategies in patients with early RA. High levels of *survivin* identify patients with a worse prognosis and a risk for disease reactivation attended by deteriorating functional disability while on MTX monotherapy, whereas the combination of synthetic disease-modifying drugs appears to be more effective than a biological treatment strategy with anti-TNF.

## Abbreviations

CI: Confidence interval
DAS28: 28-joint count disease activity score
HAQ: Health Assessment Questionnaire
HCQ: Hydroxychloroquine
HLA: Human leukocyte antigen
IL: Interleukin
IQR: Interquartile range
MTX: Methotrexate
OR: Odds ratio
PtGA: Patient’s global assessment of disease activity
RA: Rheumatoid arthritis
RF: Rheumatoid factor
SSZ: Sulfasalazine
SWEFOT trial: Swedish pharmacotherapy trial
Th: T helper cells
TNF: Tumor necrosis factor
TT: Triple therapy
VAS: Visual analog scale
WHO: World Health Organization
